# Epicardial fat tissue, a hidden enemy against the early recovery of left ventricular systolic function after transcatheter aortic valve implantation

**DOI:** 10.1016/j.ijcha.2024.101595

**Published:** 2025-01-03

**Authors:** Helen S. Anwar, Pilar Lopez Santi, Magdy Algowhary, Mohamed Aboel-Kassem F. Abdelmegid, Hatem A. Helmy, J. Wouter Jukema, Nina Ajmone Marsan, Frank Van Der Kley

**Affiliations:** a Department of Cardiovascular Medicine, Assiut University Heart Hospital, Assiut University, Assiut, Egypt; b Department of Cardiology, Leiden University Medical Center, Leiden, the Netherlands

**Keywords:** Epicardial fat tissue, Global longitudinal strain, Severe aortic stenosis, Transcatheter aortic valve implantation

## Abstract

**Background:**

Epicardial fat tissue (EFT) is an active organ that can affect cardiac function and structure through endocrine, paracrine, and proinflammatory mechanisms. We hypothesized that greater thickness of EFT may harm the recovery of left ventricular (LV) systolic function in patients with severe aortic stenosis (AS) and reduced LV ejection fraction (EF ≤ 50 %) undergoing transcatheter aortic valve implantation (TAVI).

**Methods:**

A sixty six patients with severe AS and 20 % ≥ LVEF ≤ 50 % who underwent TAVI were included. Patients were categorized into two groups based on LV systolic function recovery 30 days after TAVI defined by ≥ 20 % relative increase in LV Global longitudinal strain (GLS) from baseline. EFT was determined by ECG-gated contrast-enhanced multidetector computed tomography (MDCT).

**Results:**

Forty-five patients (68.0 %) showed LV systolic function recovery. EFT showed no significant correlation with the baseline LV-GLS but was associated with less likelihood of LV systolic function recovery (OR 0.7, 95 % CI 0.50 – 0.98, P = 0.04). In the multivariate analysis, higher LVMI (OR 1.05, 95 % CI 1.00–1.10, P = 0.02), lower LV-GLS (OR 0.55, 95 % CI 0.40–0.82, P = 0.002), and thinner EFT (OR 0.38, 95 % CI 0.20–0.73, P = 0.003) were independently associated with LV systolic function recovery after TAVI.

**Conclusion:**

EFT extent is associated with LV systolic function recovery in AS patients with impaired LVEF undergoing TAVI and therefore may help in risk stratification and management of these patients.

## 1 Introduction

Aortic stenosis (AS) is the most prevalent primary valve disorder among older adults in Western countries, placing a significant burden on healthcare systems [1]. AS affects not only the aortic valve but also leads to left ventricular (LV) remodeling, fibrosis, and ultimately LV systolic dysfunction [2].

Studies show that nearly one-third of patients with severe AS experience LV systolic dysfunction, with two-thirds showing improvement after transcatheter aortic valve implantation (TAVI) [3], [4]. Recent studies strongly suggest speckle-tracking-derived Global longitudinal strain (GLS), as a more sensitive and accurate method to identify LV functional improvement after TAVI. A relative increase in LV-GLS of ≥ 20 % as compared to baseline was proposed as a threshold of a significant LV systolic function recovery [5], [6].

Epicardial fat tissue (EFT) is a metabolically active fat depot between the epicardium and visceral pericardium. It can influence cardiac structure and function through endocrine, paracrine, and proinflammatory mechanisms [7]. Eventually, EFT is considered a promising marker for risk stratification in cardiovascular disease [8].

In severe AS patients undergoing TAVI, EFT has been linked to short- and medium-term outcomes [9], [10]. The interplay between EFT and LV systolic function in such patients remains underexplored. This study aims to investigate the association between EFT and LV systolic function recovery post-TAVI in patients with severe AS and reduced LV ejection fraction (LVEF ≤ 50 %).

## 2 Methods

### 2.1 Patient population and data collection

We retrospectively reviewed 974 patients with severe AS who underwent TAVI between January 2019 and December 2023 at the Leiden University Medical Center, Leiden, the Netherlands. We included only patients with reduced LV systolic function (20 % ≤ LVEF ≤ 50 %) and had none of our exclusion criteria (previous open-heart surgery for coronary artery bypass (CABG) or valve replacement, prior percutaneous coronary intervention (PCI) due to ST-elevation myocardial infarction (STEMI) or non-ST elevation myocardial infarction (NSTEMI), concomitant severe mitral or aortic valve regurgitation). Patients with poor MDCT quality were excluded. Our study finally included 66 patients. All patients underwent transthoracic echocardiogram (TTE), MDCT, and coronary angiography before TAVI. 30 days after TAVI, a TTE was done as a routine follow-up. The departmental electronic medical record (EPD-vision 12.14.0.1; Leiden University Medical Center, Leiden, the Netherlands) was used to collect all demographic and clinical data. The Scientific Institutional Review Board approved this retrospective analysis.

### 2.2 TTE data acquisition and measurements

TTE was conducted using Vivid-7, E9, or E95 systems (General Electric Vingmed, Horten, Norway). Two-dimensional, M−mode, color, continuous-wave, and pulsed-wave Doppler images were captured from parasternal, apical, and subcostal views. Offline echocardiographic analysis was performed with EchoPac software (version 204; GE Medical Systems, Horten, Norway). Peak and mean transaortic gradients were calculated from apical 3- or 5-chamber views using continuous-wave Doppler and the Bernoulli equation. Aortic valve area (AVA) was estimated via the continuity equation and normalized to body surface area (indexed AVA). Severe AS was defined as an AVA less than 1.0 cm^2^ or indexed AVA below 0.6 cm^2^/m^2^, combined with a mean transaortic pressure gradient of at least 40 mm Hg or a peak aortic jet velocity of 4 m/s or higher [11].

Stroke volume (SV) and stroke volume index were measured via pulsed-wave Doppler of the left ventricular outflow tract at apical 3- or 5-chamber view. Based on these parameters, severe AS can be categorized hemodynamically as follows:

• High gradient AS, characterized by a mean pressure gradient of at least 40 mm Hg, irrespective of SV index value.
• Low-flow, low-gradient AS, defined by a mean pressure gradient below 40 mm Hg and an SV index ≤ 35 mL/m^2^ [12].

LV end-diastolic and end-systolic volumes were measured in the apical 2- and 4-chamber views and LVEF was calculated via Simpson's biplane method. LV dimensions, including intraventricular septal thickness, LV end-diastolic diameter, and posterior wall thickness, were assessed in the parasternal long-axis view during end-diastole. LV mass was calculated using the Devereux formula and adjusted to the body surface area [13].

Aortic, mitral, and tricuspid valve regurgitation severity were identified and graded according to current recommendations [14].

### 2.3 Speckle-tracking echocardiographic examination

**Fig. 1:**
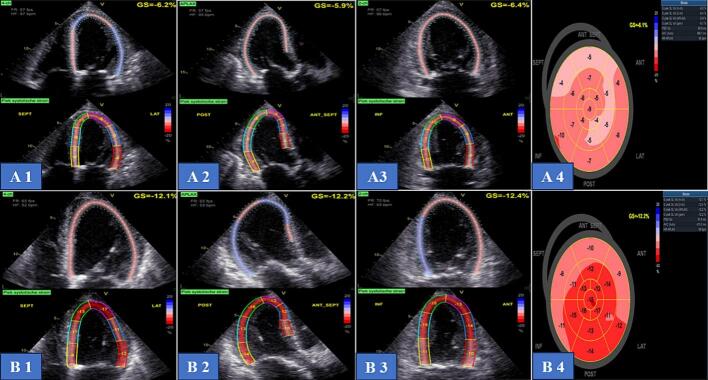
**Example of a patient who showed LV systolic function recovery after TAVI: A,** LV-GLS before TAVI = −6.1 %, **B,** 30 days after TAVI LV-GLS = − 12.2 % so the relative increase in LV-GLS = 100 %.

The LV-GLS was assessed offline using two-dimensional speckle-tracking echocardiography with EchoPac software (version 204, GE Vingmed Ultrasound, Horten, Norway). LV-GLS was calculated based on images from the apical 4-, 3-, and 2-chamber views, zoomed on the LV at a frame rate of ≥ 50 frames per second. The LV endocardial border was automatically traced (with manual correction where needed) and tracked throughout the cardiac cycle using the software. The LV-GLS was determined by averaging the peak strain values across all segments from the apical views and presented as negative values. (Fig. 1 A & B). More negative values correspond to better LV systolic function [13].

### 2.4 MDCT acquisition and analysis

MDCT-specific acquisition TAVI protocol was performed using a 320 MDCT scanner (Aquilion ONE, Toshiba Medical Systems, Otawara, Japan), allowing anatomical assessment of the AV, aortic root and arch, and the aorto-iliofemoral vasculature. Prospective ECG gated data acquisition for the thorax (the heart and aortic root) with a slice thickness of 0.5 mm then non-ECG gated acquisition of the abdomen and pelvis with a slice thickness of 2.0 mm then image reconstruction was done at different percentages of the cardiac cycle (R-R interval) [15]. Data processing was performed in a remote workstation with dedicated CT analysis software (Sectra workstation IDS7, Version 24.1.10.5437).

**Fig. 2:**
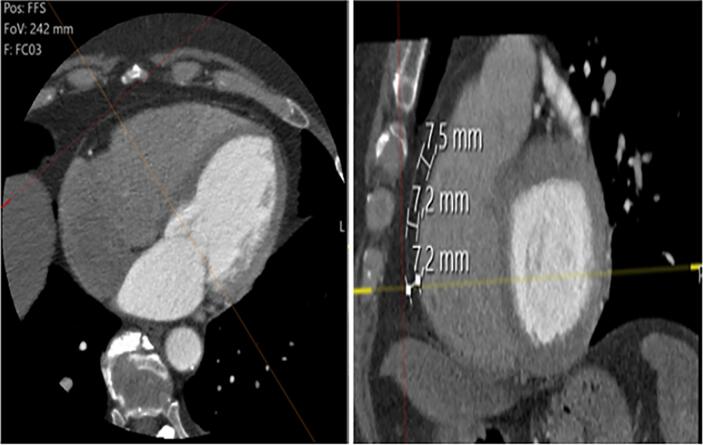
**Quantification of EFT thickness on MDCT:** EFT is defined as fat epicardium and the visceral pericardium at the basal level of the short-axis images. Three measurements were taken at the upper, middle, and lower levels (75, 50, and 25% level of full length, respectively) of the right ventricle.

Using the same ECG-gated reconstructed images. EFT was measured between the epicardium and the visceral pericardium at the basal level of the short-axis images. Three measurements were taken at the right ventricle's upper, middle, and lower levels (75, 50, and 25 % level of full length, respectively) (Fig. 2). The average of the three measurements was used for the analysis [16].

### 2.5 Coronary angiography

Coronary angiography was done as a routine institutional protocol for all TAVI candidate patients before the procedure and PCI was performed for patients with significant coronary artery disease (coronary stenosis > 70 % in a major epicardial coronary artery) [12].

### 2.6 TAVI procedure

TAVI decision-making of the access route and valve type and size was obtained by the local heart team based on the pre-TAVI MDCT data analysis. TAVI procedure was performed according to standard practice under local anesthesia via transfemoral or trans axillary vascular arterial access using either balloon-expandable valves (Edwards Lifesciences SAPIEN 3/ 3 ultra) or self-expanding valves (Medtronic Evolut PRO/ PRO + and Boston Scientific Accurate Neo2).

### 2.7 30 days follow-up TTE

A TTE was done for all patients 30 days after TAVI using the same protocol previously mentioned in the pre-TAVI evaluation.

LV systolic function recovery was defined by a ≥ 20 % relative increase in LV-GLS as compared with the baseline [6]. Accordingly, patients were categorized into two distinct groups either with or without LV systolic function recovery.

### 2.8 Statistical analysis

Shapiro-Wilk test was used to test the normality of data. Continuous data are presented as mean ± standard deviation (SD) if normally distributed and median ± interquartile range (IQR) if not normally distributed. Categorical data are presented as absolute numbers and percentages. Paired T-test and Mc Nemar Bowker test were used to compare the continuous and categorical variables before and after TAVI. The independent sample T-test and ANOVA test were used to compare continuous variables between groups while the chi-square test was used to compare categorical variables. The Spearman correlation test was used to identify the correlation between different variables and EFT. Uni-and multivariate binary logistic regression models were done for possible LV systolic function recovery associators.

All statistical analyses were performed with SPSS software version 29.0 (IBM, Armonk, NY). Significant variables in the univariate logistic regression with p-value < 0.05 were entered in a multivariate logistic regression analysis to obtain the independent predictors of LV function recovery (odds ratio (OR), 95 % confidence interval (CI)). A 2-tailed p-value less than 0.05 is considered statistically significant.

## 3 Results

### 3.1 Baseline patient characteristics

A total of 66 patients with severe symptomatic AS and LV dysfunction (20 %≤ LVEF ≤ 50 %) underwent TAVI and met the criteria for our study. The baseline characteristics of our study population are shown in Table 1. The mean age was 82.6 ± 8.9 years. 60.6 % of the participants were males, 66.6 % had arterial hypertension and 80.3 % had no coronary artery disease. Regarding rhythm, 37 patients were in sinus rhythm, 22 had AF, and 7 had permanent pacemakers. 75.7 % of our study population had high surgical risk with logistic EuroSCORE ≥ 10 %. Table 2 displays the baseline TTE, MDCT, and procedural data of the study population, the mean transaortic mean pressure gradient was 43.0 ± 12.5 mmHg while the mean of AVA was 0.76 ± 0.20 cm^2^. 59.1 % of our patients had high-gradient severe aortic stenosis. The mean baseline LVEF was 35.4 ± 6.9 % and the mean baseline LV-GLS was −8.9 % ± 3.2 %. The mean LVMI was 146.0 ± 34.1 g/m^2^. The median (IQR) EFT thickness was 4.0 (2.9–––5.5) mm.

**Table 1:** Comparison of baseline demographic and laboratory data of enrolled patients:

**Variables**	**All patients** **N = 66**	**LV recovery** **Yes** **(N = 45)**	**LV recovery** **No** **(N = 21)**	**P value**
Age (years) ± SD	82.6 ± 8.9	83.1 ± 9.1	81.7 ± 8.5	0.55
Male sex, n (%)	40 (60.6 %)	26 (57.8 %)	14 (66.7 %)	0.49
Body mass index (kg/m^2^), ±SD	26.5 ± 5.5	26.6 ± 5.9	26.5 ± 4.7	0.94
Hypertension, n (%)	44 (66.6 %)	26 (57.8 %)	18 (85.7 %)	**0.02**
Diabetes mellitus, n (%)	20 (30.3 %)	12 (26.7 %)	8 (38.1 %)	0.80
**CAD**				0.43
No CAD, n (%)	53 (80.3 %)	38 (84.4 %)	15 (71.5 %)	
One vessel, n (%)	8 (12.1 %)	4 (8.9 %)	4 (19.0 %)	
Two vessels, n (%)	5 (7.6 %)	3 (6.7 %)	2 (9.5 %)	
**ECG**				**0.003**
Sinus rhythm, n (%)	37(56.1 %)	31 (68.9 %)	6 (28.5 %)	
AF, n (%)	22(33.3 %)	9 (20.0 %)	13 (62.0 %)	
Pacemaker, n (%)	7(10.6 %)	5 (11.1 %)	2 (9.5 %)	
Pre TAVI LBBB, n (%)	22 (33.3 %)	15 (28.8 %)	8 (34.8 %)	0.60
**Laboratory data**				
Hemoglobin (mmol/l) ± SD	7.7 ± 0.9	7.7 ± 0.9	7.5 ± 0.9	0.41
Estimated Glomerular filtration rate (ml/minute//1.73 m^2^) ± SD	55.8 ± 20.3	58.1 ± 18.4	51.1 ± 23.6	0.19
Logistic Euro SCORE I %	13.8 %	13.8 %	18.6 %	0.5
(median, IQR)	(10.1–20.6)	(9.6–19.2)	(10.3–24.8)	
High surgical risk ≥ 10 %, n (%)	50 (75.7 %)	36 (80.0 %)	14 (66.6 %)	

Data was expressed in the form of mean ± SD, frequency (percentage), and median (IQR), p-value is significant if < 0.05. **AF:** atrial fibrillation**, CAD**: coronary artery disease, **ECG**: electrocardiogram, **LBBB**: left bundle branch block.

**Table 2:** Comparison of baseline TTE, MDCT, and procedural data among enrolled patients.

**Variables**	**All patients** **N = 66**	**LV recovery** **Yes** **(N = 45)**	**LV recovery** **No** **(N = 21)**	**P value**
Mean transaortic pressure gradient (mmHg) ±SD	43.0 ± 12.5	45.3 ± 11.4	38.4 ± 13.5	**0.03**
Transaortic maximum velocity (m/s) ±SD	3.9 ± 0.6	3.9 ± 0.5	3.8 ± 0.6	0.19
Aortic valve area (cm) ±SD	0.76 ± 0.20	0.72 ± 0.18	0.85 ± 0.14	**0.007**
Indexed Aortic valve area (cm/m^2^) ±SD	0.4 ± 0.1	0.37 ± 0.09	0.44 ± 0.11	**0.02**
Stroke volume index (ml/m^2^) ±SD	33.9 ± 7.7	33.6 ± 6.3	33.8 ± 7.0	0.5
**AS type**				**0.004**
High gradient, n (%)	39(59.1%)	32(71.1%)	7 (33.3%)	
LFLG, n (%)	27(40.9%)	13(28.9%)	14 (66.6%)	
**Ejection fraction (%)** ±SD	35.4 ± 6.9	34.1 ±5.6	38.9 ±7.7	**0.002**
**Ejection fraction grading**				**0.04**
21- 30 %, n (%)	16(24.2%)	13 (28.9%)	1 (4.8%)	
31- 40 %, n (%)	36(54.5%)	24 (53.3%)	12 (57.1%)	
41- 50 %, n (%)	16(17.8%)	8 (17.8%)	8 (38.1%)	
Global longitudinal strain % ±SD	-8.9% ± 3.2	-8.3 % ± 1.5	-8.5 % ± 2.1	**<0.001**
LAVI (ml/m^2^) ±SD	48.3 ± 11.5	49.6±10.1	45.8±13.8	0.21
IVSD (mm) ±SD	12.3 ± 1.5	12.2 ± 1.5	12.3 ± 1.4	0.89
LVEDD (mm) ±SD	54.8 ± 6.8	55.9 ± 7.1	52.5 ± 5.5	0.05
LVESD (mm) ±SD	44.9 ± 6.9	46.3± 7.5	42.3 ± 4.7	0.02
LVMI (g/m^2^) ±SD	146.0± 34.1	155.1±36.2	128.8±19.9	**<0.001**
**AR grade**				0.37
No, n (%)	33 (50.0 %)	20 (44.4 %)	13 (61.9 %)	
Mild, n (%)	23 (34.8 %)	18 (40.0 %)	5 (23.8 %)	
Moderate, n (%)	10 (15.2 %)	7 (15.6 %)	3 (14.3 %)	
**MR grade**				0.71
Mild, n (%)	30 (45.5 %)	20 (44.4 %)	10 (47.6 %)	
Moderate, n (%)	29 (43.9 %)	21 (46.7 %)	8 (38.1 %)	
Moderately severe, n (%)	7 (10.6 %)	4 (8.9 %)	3 (14.3 %)	
**TR grade**				0.63
Mild, n (%)	42 (78.7 %)	30 (66.6 %)	12 (57.1 %)	
Moderate, n (%)	20 (30.3 %)	12 (26.7 %)	8 (38.1 %)	
Severe, n (%)	4 (6.0 %)	3 (6.7 %)	1 (4.8 %)	
**Type of native aortic valve**				0.38
Tricuspid, n (%)	56(84.8 %)	37 (82.8 %)	19 (90.5 %)	
Bicuspid, n (%)	10(15.2 %)	8 (17.8 %)	2 (9.5 %)	
**TAPSE**	18.7 ± 2.7	18.9 ± 2.5	18.3 ± 3.1	0.4
EFT thickness (mm), median (IQR)	4(2.9–5.5)	3.5(2.8–5.1)	4.6(3.3–6.0)	**0.03**
**Type of TAVI valve**				0.39
SAPIEN 3/3 ultra, n (%)	50(75.8 %)	32(71.1 %)	18 (85.7 %)	
Evolut pro/pro+, n (%)	15(22.7 %)	12 (26.7 %)	3 (14.3 %)	
Accurate Neo 2, n (%)	1(1.5 %)	1 (2.2 %)	0 (0.0 %)	
**Final PVL**				0.95
Mild and less, n (%)	63(95.5 %)	43 (95.6 %)	20 (95.2 %)	
Moderate, n (%)	3(4.5 %)	3 (4.4 %)	1 (4.8 %)	

Data was expressed in the form of mean ± SD, median (IQR), and frequency (percentage). p-value is significant if < 0.05. **AR**: aortic regurgitation, **AS**: aortic stenosis, **EFT**: epicardial fat tissue, **IVSD**: interventricular septum diastolic diameter, **LFLG**: low flow low gradient, **LAVI**: left atrial volume index, **LVEDD**: left ventricular end-diastolic dimension, **LVESD**: left ventricular end-systolic dimension, **LVMI**: left ventricular mass index, **MR**: mitral regurgitation, **PVL**: paravalvular leakage, **TAPSE:** tricuspid annular plane systolic excursion, **TR**: tricuspid regurgitation.

### 3.2 TTE follow‑up at 30 days post-TAVI

Significant hemodynamic improvement was noticed after TAVI, Table 3 showed TTE findings before and 30 days after TAVI. A statistically significant improvement in LVEF as compared to baseline was observed in the 30 days follow-up (35.5 % ± 6.9 % to 46.6 % ± 7.1 %, P < 0.001), and a statistically significant improvement of LV-GLS (−8.9 % ± 3.2 to −11.1 % ± 2.9 %, P < 0.001). LVMI also showed a statistically significant reduction compared to the baseline (146.0 ± 34.1 g/m^2^ to 133.3 ± 28.6 g/m^2^, P < 0.001). 30 days after TAVI, 45 (68.0 %) patients showed a ≥ 20 % relative increase in LV-GLS as compared to baseline. Accordingly, patients were categorized into two groups either with or without LV systolic function recovery. Table 1, Table 2 show the comparison between the two groups, baseline characteristics were similar apart from 85.7 % of patients in the without recovery group were hypertensive (P = 0.02). A total 62.0 % of patients in the group without recovery had AF, while AF was representative in only 20 % of patients in the recovered group (P = 0.003). Regarding the TTE findings, patients with LV systolic function recovery had a baseline higher transaortic mean pressure gradient (45.3 ± 11.4 mmHg vs 38.4 ± 13.5 mmHg), smaller AVA (0.72 ± 0.18 cm vs 0.85 ± 0.14 cm), higher LVMI (155.1 ± 36.2 gm/m^2^ vs 128.8 ± 19.9 gm/m^2^) than patients without LV systolic function recovery (P = 0.03, 0.007, <0.0001, respectively). Regarding LV systolic function, patients in the recovered group had statistically significant lower baseline LV-GLS (−8.3 % ± 1.5 % vs −8.5 % ± 2.1 %) and lower baseline LVEF (34.1 ± 5.6 % vs 38.9 ± 7.7 %) than patients without LV systolic function recovery (P < 0.001, 0.002, respectively). A total of 24 of the 36 patients with LVEF 31––40 % showed recovery of LV systolic function after TAVI. In the context of MDCT and procedural data, there was no difference between the two groups except for EFT which showed a statistically significant lower thickness in patients with LV systolic function recovery group than in patients without LV systolic function recovery (P = 0.03).

**Table 3:** TEE findings before and 30 days follow up after TAVI in the enrolled patients.

**Variable**	**Before TAVI**	**After TAVI**	**P value**
Mean pressure gradient (mmHg) ±SD	43.0 ± 12.5	9.1 ± 3.8	**0.03**
Ejection fraction % ±SD	35.5 % ± 6.9	46.6 % ± 7.1	**<0.001**
LV-GLS % ±SD	-8.9 % ± 3.2	-11.1% ± 2.9	**<0.001**
LAVI (ml/m^2^) ±SD	48.3 ± 11.5	42.6 ± 9.2	**<0.001**
LVMI (g/m^2^) ±SD	146.0 ± 34.1	133.3 ± 28.6	**<0.001**
**MR grade**			**0.04**
Mild, n (%)	30 (45.5 %)	46 (69.6 %)	
Moderate, n (%)	29 (43.9 %)	17 (25.8 %)	
Moderately severe, n (%)	7 (10.6 %)	3 (4.5 %)	
**TR grade**			**0.03**
Mild, n (%)	42 (78.7 %)	55 (83.3 %)	
Moderate, n (%)	20 (30.3 %)	9 (13.6 %)	
Severe, n (%)	4 (6.0 %)	2 (3.0 %)	
PASP (mmHg) ±SD	38.7 ± 10.3	32.4 ± 7.5	**<0.001**
TAPSE (mm) ±SD	18.7 ± 2.7	19.0 ± 2.6	**<0.001**

Data was expressed in the form of mean ± SD and frequency (percentage). P-value is significant if < 0.05. **AR**: aortic regurgitation **GLS:** global longitudinal strain**, LAVI**: left atrial volume index, **LVMI**: left ventricular mass index, **MR**: mitral regurgitation, **PASP**: pulmonary artery systolic pressure, **TAPSE:** tricuspid annular plane systolic excursion, **TR**: tricuspid regurgitation.

### 3.3 EFT and LV systolic function

A statistically significant positive correlation existed between EFT and weight and BMI (r = 0.28, 0.39, and P = 0.02, 0.001, respectively). Considering the TTE parameters, our study found no significant correlation between EFT and baseline LV systolic function assessed by both LVEF and LV-GLS, Table 4.

**Table 4:** Correlation between EFT thickness and baseline demographic and TTE derived parameters of patients:

**Variables**	**r (P value)**
Age (years)	−0.12 (0.3)
Weight (kg)	**0.28 (0.02)**
BMI (kg/m2)	**0.39 (0.001)**
LVMI (gm/m^2^)	0.07 (0.5)
LAVI (ml/ m^2^)	0.07 (0.5)
LVESD (mm)	0.17 (0.2)
LVEDD (mm)	0.10 (0.4)
Baseline LVEF (%)	0.002 (0.9)
Baseline LV-GLS (%)	−0.18 (0.2)

Data was expressed in the form of *r* (indicates the strength of correlation) and p-value (indicates the significance of correlation if < 0.05)**. BMI**: body mass index, **LAVI:** left atrial volume index, **LVEDD**: left ventricular end-diastolic dimension, **LVEF**: left ventricular ejection fraction, **LVESD**: left ventricular end-systolic dimension, **LV-GL**S: left ventricular global longitudinal strain, **LVMI**: left ventricular mass index.

In the analysis of factors associated with early LV systolic function recovery, univariable binary logistic regression identified that patients without AF, with smaller AVA, higher LVMI, lower baseline LVEF, reduced baseline LV-GLS, and thinner EFT were more likely to experience LV systolic function recovery. In the multivariate analysis, due to model constraints, two separate models were presented. In model 1, higher LVMI (OR 1.05, 95 % CI 1.00–1.10, P = 0.02), lower LV-GLS (OR 0.55, 95 % CI 0.40–0.82, P = 0.002), and thinner EFT (OR 0.38, 95 % CI 0.20–0.73, P = 0.003) were independent predictors of LV function recovery post-TAVI. In model 2, absence of AF (OR 0.16, 95 % CI 0.03–0.88, P = 0.03), higher LVMI (OR 1.05, 95 % CI 1.00–1.10, P = 0.02), lower LV-GLS (OR 0.55, 95 % CI 0.37–0.81, P = 0.002), and thinner EFT (OR 0.37, 95 % CI 0.19–0.74, P = 0.005) remained significant predictors, Table 5.

**Table 5:** Univariate and multivariate binary logistic analysis of the baseline associates of LV systolic function recovery following TAVI:

	**Univariate analysis**				**Multivariate analysis** **Model 1**			**Multivariate analysis** **Model 2**		
**Variables **		**Odd’s ratio**	**95** % **confidence interval**	**P** **value**	**Odd’s ratio**	**95** % **confidence interval**	**P value**	**Odd’s ratio**	**95** % **confidence interval**	**P value**
Age		1.0	0.96–1.07	0.55	−	−	−	−	−	−
Female		1.46	0.49–4.32	0.49	−	−	−	−	−	−
BMI		1.01	0.92–1.12	0.75	−	−	−	−	−	−
Estimated GFR ml/min/1.73 m^2^		1.02	0.99–1.04	0.21	−	−	−	−	−	−
Hgb (mmol/l)		1.39	0.76–2.54	0.28	−	−	−	−	−	−
AF		0.14	0.04–0.45	**<0.001**	−	−	−	0.16	0.03–0.88	**0.03**
Pre-TAVI LBBB		1.37	0.45–4.25	0.56	−	−	−	−	−	−
CAD		0.46	0.13–1.59	0.22	−	−	−	−	−	−
PG mean		1.06	1.01–1.12	**0.02**	0.008	0.00–1.45	0.06	−	−	−
AVA		0.01	0.00–0.37	**0.01**	−	−	−	−-	−	−
LVMI (gm/m^2^)		1.03	1.01–1.06	**0.003**	1.05	1.00–1.10	**0.02**	1.05	1.00–1.10	**0.02**
LVEF (%)		0.86	0.78–0.95	**0.004**	−	−	−	−	−	−
LV-GLS (%)		0.63	0.48–0.81	**<0.001**	0.55	0.40–0.82	**0.002**	0.55	0.37–0.81	**0.002**
EFT-thickness (mm)		0.70	0.50–0.98	**0.04**	0.38	0.20–0.73	**0.003**	0.37	0.19–0.74	**0.005**

**AVA:** Aortic valve area, **AF:** atrial fibrillation, **BMI**: body mass index, **CAD:** coronary artery disease, **EFT:** epicardial fat tissue, **GFR:** glomerular filtration rate, **LBBB**: left bundle branch block, **LVEF:** left ventricular ejection fraction, **LV-GLS** left ventricular global longitudinal strain, **PG mean**: transaortic mean pressure gradient, **TAVI:** transcatheter aortic valve implantation.

## 4 Discussion

Our study investigated the early LV systolic function recovery in a subgroup of patients with severe AS and reduced LV systolic function undergoing TAVI focusing on the EFT as a newly proposed cardiovascular risk factor. The Key findings are as follows: (1) Nearly two-thirds of this group of patients had early LV systolic function improvement post-TAVI, and this improvement was more obvious in patients with baseline LVEF 31–40 % compared to other LVEF ranges, (2) Higher LVMI, lower LV-GLS, and thinner EFT were associated with early LV systolic function improvement after TAVI.

Our study showed that 68.0 % of our patients had early LV systolic function recovery after TAVI. The largest percentage of recovery was noted in patients with a baseline LVEF of 31–40 % which is consistent with data from a previous study [17].

LV systolic function recovery occurs significantly in patients with higher baseline transaortic mean gradient and this is matching with the results from the PARTNER trial and other studies [3], [4]. Also, sinus rhythm and smaller AVA were noticed with statistically significant differences in the recovered group and the same observation was given by Kolte et al [18].

### 4.1 EFT and LV systolic function recovery after TAVI

EFT represents almost 20 % of the human heart mass. Under physiological circumstances, it has a cardioprotective function through biochemical, thermogenic, and mechanical properties. EFT provides the heart with free fatty acids (FFA), the main energy substrate for cardiomyocyte contractility [19]. However, the excess EFT may have an unfavorable effect on the heart through the synthesis and secretion of proinflammatory and proatherogenic factors called adipocytokines that cause inflammation and microvascular dysfunction and directly impair the myocardial function [20], [21]. Increased EFT has been associated with direct infiltration and accumulation of fat cells (adipocyte) in the ventricular myocardium which in turn leads to inflammation, oxidative stress, and finally myocardial apoptosis with interstitial fibrosis [22], [23]. Accordingly, EFT has been identified as a risk factor in the pathogenesis and progression of coronary artery disease and atrial fibrillation [24].

The interaction between EFT and LV systolic function is still a matter of controversy, and still a subject of research. Some studies showed larger EFT volumes were associated with reduced LV myocardial contractility [23], while others found that smaller EFT volumes in patients with reduced LV systolic function [25]. A newly published study for patients with non-ischemic cardiomyopathy concluded that better LV reverse remodeling was seen with high EFT volumes [26]. This conflict in the studies' results is suggestive of an overly complex interaction between the regulatory pathways and properties of EFT and the myocardium between the different patient populations.

In patients with severe AS, our study found no significant correlation between EFT thickness and baseline LV systolic function. However, EFT was significantly thicker in patients without LV systolic function recovery than those with recovered systolic function after TAVI (P = 0.03) with less likelihood of LV systolic function recovery in the presence of thicker EFT as shown by the univariate analysis (P = 0.04). The proposed deleterious effect of excessive EFT on LV myocardial structure and function can be assigned to augmentation of multiple factors such as obesity, coronary artery disease and AF not EFT itself only.

Considering the predictors of LV systolic function recovery after TAVI, our 2 models of multivariate logistic regression demonstrated as significant the same 3 variables: (1) greater LVMI was independently associated with LV systolic function recovery which is in line with the findings of the study by Y. Jeong et al, [27]. (2) lower baseline LV-GLS was found to be independently associated with recovery of LV systolic function. (3) thicker EFT was inversely associated with recovery.

A recent study showed that patients with higher EFT had a significantly higher 5-year all-cause mortality compared to patients with low EFT and this was independent of other cardiovascular risk factors; therefore by adding such a new easily calculated parameter, we can improve the selection of patients that would benefit from interventional therapies and enhance the outcomes of TAVI [28]. Taking into consideration the patient rhythm, our second module showed that patients with AF were > 2.0 × less likely to have early LV systolic function recovery than those with sinus and this observation can be explained by the known effect of longstanding tachyarrhythmia especially AF in the LV systolic dysfunction (tachycardia-induced cardiomyopathy) [29], and this finding is in line with the results of Kuneman et al, study [17].

The strengths of our study include the following: Firstly, we use LV-GLS, which more precisely and accurately identifies the minimal and subclinical changes for assessment of LV systolic function and investigating the recovery status. Secondly, our study applied rigorous exclusion criteria that could influence LV systolic function; eliminating these confounders enhances our findings' accuracy.

### 4.2 Study limitations

The relatively small sample size limits the possibility of correcting for confounders. The retrospective cohort design of the research precludes the investigation of direct causal relationships between epicardial fat, comorbid conditions, and myocardial function and contractility. Lack of blinded core lab assessment can introduce observer and measurement bias.

## 5 Conclusion

Less EFT thickness is positively associated with LV systolic function recovery in patients with severe AS and reduced LVEF after successful TAVI.

Clinical Implications:

EFT, a marker of visceral adiposity, negatively affects myocardial structure and function, potentially influencing LV systolic function recovery post-TAVI. Therefore, targeting EFT reduction may improve post-TAVI outcomes. Weight loss has been shown to reduce visceral adiposity, including EFT [30]. Additionally, small studies suggest medications like sodium-glucose cotransporter-2 inhibitors (e.g., dapagliflozin) may reduce EFT and improve LV systolic function in patients with type 2 diabetes [31]. Future large-scale studies are needed to further evaluate the impact of EFT reduction on myocardial function.

## Ethical approval

Ethical approval was waived by the local Ethics Committee of Leiden University Medical Center (LUMC) because of the study's retrospective nature and all the procedures being performed were part of routine care.

## CRediT authorship contribution statement

**Helen S. Anwar:** Writing – original draft, Methodology. **Pilar Lopez Santi:** Writing – review & editing, Methodology. **Magdy Algowhary:** Writing – review & editing. **Mohamed Aboel-Kassem F. Abdelmegid:** Writing – review & editing. **Hatem A. Helmy:** Writing – review & editing. **J. Wouter Jukema:** Writing – review & editing. **Nina Ajmone Marsan:** Writing – review & editing. **Frank Van Der Kley:** Writing – review & editing.

## Funding

The present article received no external funding.

## Declaration of competing interest

The authors declare the following financial interests/personal relationships which may be considered as potential competing interests: The Department of Cardiology of the Leiden University Medical Center received unrestricted research grants from Abbot Vascular, Bayer, Biotronic, Boston Scientific, Edwards Lifesciences, GE Healthcare, Medtronic, and Novartis. The remaining authors have no conflicts of interest to disclose.
